# Research on Progress in Combined Remediation Technologies of Heavy Metal Polluted Sediment

**DOI:** 10.3390/ijerph16245098

**Published:** 2019-12-13

**Authors:** Min Zhang, Xiangchun Wang, Long Yang, Yangyang Chu

**Affiliations:** China Urban Construction Design & Research Institute Co. Ltd., Beijing 100120, China; zhangmin@cucd.cn (M.Z.); wangxiangchun@cucd.cn (X.W.); chuyangyang@pku.edu.cn (Y.C.)

**Keywords:** sediment, heavy metal pollution, combined remediation technology

## Abstract

Heavy metals contaminated sediment has become a worldwide environmental issue due to its great harm to human and aquatic organisms. Thus, economical, effective, and environmentally-friendly remediation technologies are urgently needed. Among which, combined remediation technologies have attracted widespread attention for their unique advantages. This paper introduces combined remediation technologies based on physical-, chemical-, and bio-remediation of heavy metal polluted sediments. Firstly, the research progress in physical-chemical, bio-chemical, and inter-organismal (including plants, animals, microorganisms) remediation of heavy metal polluted sediments are summarized. Additionally, the paper analyzes the problems of the process of combined remediation of heavy metals in river sediments and outlooks the future development trends of remediation technologies. Overall, this review provides useful technology references for the control and treatment of heavy metal pollution in river sediments.

## 1. Introduction

Heavy metals entering water bodies will be accumulated in the riverbed surface sediment through adsorption, complexation, precipitation, etc. Therefore, heavy metal concentrations in sediments are far higher than that in the overlying water. What is worse, once the environmental conditions change, accumulated heavy metals can be released from the sediment through desorption, dissolution, oxidation, and reduction, causing secondary pollution to the water body [1]. Continuous accumulation and migration of heavy metals in sediment will not only pose a serious threat to aquatic organisms and irrigation of farmland along rivers, but also endanger human health via drinking water or food chains [2]. Therefore, treatment and control of heavy metal pollution in sediment should be stressed. These following three aspects are the keys of remediation of heavy metal polluted sediment: (1) eliminate or reduce the environmental toxicity of heavy metals in the sediment; (2) transform the polluted sediment into harmless substances; and (3) isolate and remove the polluted sediment from the water body. Moreover, solving these core problems is of great importance to achieve better treatment and control of the polluted sediment and fine purification of river ecology.

At present, the remediation methods of the heavy metal polluted sediment mainly include physical, chemical, and biological methods. Remediation of heavy metals with a single method may lead to problems such as low efficiency and incomplete remediation, especially in the case of combined pollution with multiple heavy metals in sediment. An integral remediation solution combining two or more remediation technologies will have special superiorities over a single method, including high efficiency and low energy consumption. Combined remediation technologies offer a new approach for remediation of the heavy metal polluted sediments in river courses. This paper summarizes the research progress in combined remediation technology of heavy metal pollution in river sediment emerging in recent years in the aim of providing references for the development and application of heavy metal polluted sediment remediation.

## 2. Combined Remediation Technology

### 2.1. Physical–Chemical Combined Remediation

#### 2.1.1. Electrokinetic Combined Remediation

Electrokinetic remediation (EKR) is a new multi-disciplinary technology which was first proposed by Acar et al. in 1993. At the early stage, EKR was mainly used for dewatering and the compaction of dams and foundations in civil works [3,4]. However, in recent years, EKR has been applied for soil, sediment, and polluted groundwater remediation, for the purpose of separating and extracting heavy metals from these contaminated areas. Compared to other technologies, EKR has the advantages of obvious effect, convenient operation, less use of chemicals, and avoidance of secondary pollution [5]. The principle of EKR is to remove pollutants by means of electromigration or electroosmosis. Under the action of the electric field, heavy metals migrate towards the electrode chambers through electrodialysis and accumulate in the vicinity of the electrodes, thus realizing the separation of heavy metals from the sediment. Finally, the heavy metals are collected by means of the collection system and concentrated for further treatment [6]. In the process of treating heavy metals with EKR, a large number of H^+^ ions generate from the anodic zone and form an acidic zone. In the meantime, a great number of OH^−^ ions generate from the cathodic zone. Heavy metals migrate from the anodic zone to the cathodic zone rapidly under the action of the electric field, making them deposit easily on the cathodic zone. Such conditions will influence the removal efficiency of the heavy metals. Luckily, the introduction of chemical enhancers can improve this phenomenon as well as enhance the removal efficiency of heavy metals in sediment. Xie et al. [7] researched the effect of cathodic pH on the remediation efficiency of heavy metal polluted sediment by acidized-EKR combined remediation technology. In their experiment, acidic cathode cleaning solution was applied to make the pore water in the sediment weak acidic and neutralize OH^−^ ions generated by the cathodes in the electrolytic cell. As a result, the alkalization of the sediment was reduced while the precipitation of heavy metals was effectively prevented. Finally, the removal efficiency of heavy metals by EKR combined with acidic solution was significantly improved. The experiment showed that the removal efficiency was the highest when the pH value of cathode cleaning solution was 5. Generally, organic acids are used as acid enhancers because most of the complexes generated from them are water-soluble and easy to decompose. On the contrary, inorganic acids are difficult to degrade and may cause secondary pollution. In addition to acidification pretreatment, application of flocculant pretreatment can also significantly enhance the removal effect of heavy metals by EKR. Yang et al. [8] used cationic polyacrylamide as a chemical enhancer to pretreat the polluted sediment in the process of treating cadmium polluted sediment by EKR technology. According to the results, the sediment flocculated after the flocculant pretreatment, resulting in an obvious sediment–water separation. Thus, larger porosity of sediment rendered better channels for ion migration, and the removal efficiency of heavy metals was significantly enhanced. Last but not least, its effect was more obvious than that of the reference experiment with citric acid for acidification pretreatment.

EKR developed rapidly and became more economically feasible in recent years. In addition to the use of chemical enhancers, researchers have also exploited a series of EKR combined methods, including EKR-bioleaching technology, EKR-PRB (permeable reactive barrier) technology, EKR-adsorption technology, and EKR-ion exchange membrane technology (Table 1). The combined application of these technologies can overcome the shortcomings of single remediation technology and significantly improve the effect of EKR on heavy metals, making it a promising remediation technology in the future.

#### 2.1.2. Combined Remediation by Chemical Leaching

Leaching is a frequently used technology which shifts heavy metals from the solid phase of the sediment to the liquid phase for the purpose of cleaning the sediment. Multiple chemicals are added to help extract heavy metals in the leaching process. The following leaching processes are commonly used: acid leaching, complexing agent leaching, and surfactant leaching [25,26,27]. In the acid leaching process, acidic solution is added to reduce the pH value of the sediment. The increment of H^+^ ions will compete for binding ligands with heavy metal ions so as to increase the bioavailability content. In the complexing agent leaching process, a chelating agent extracts heavy metals from sediment by forming water-soluble chelates, which leach out heavy metals from the sediment. Particularly, chelating agents with strong complexation capability should be added for treatment of the metal ions that bind closely with the sediment via coordination bonds or covalent bonds. In the surfactant leaching process, the surfactant is applied on the surface of the sediment to remove the heavy metals from the sediment though integration with the metals. During the chemical leaching process, it is a key to select a feasible extractant which can be used to extract heavy metals without damaging the structure of the effective components in the sediment. In fact, it is very difficult to find a perfect one, because any improper addition of an extractant may result in secondary pollution. In practice, researchers try remove heavy metals from sediment by a combined remediation using multiple eluents, which can not only reduce the application quantity of eluents and reduce the risk of secondary pollution, but also effectively improve the efficiency of heavy metal removal. Yuan et al. [28] used the mixture of surfactant Triton X-100 and chelating agent ethylenediaminetetraacetic acid (EDTA) to remove heavy metals from sediment. The results showed that the removal rate of Zn reached 30.0% by utilizing the mixed solution, which increased 2%–5% compared with cases only using EDTA. Shang [29] chose chelating agent ethylenediamine N,N′-disuccinic acid (EDDS), surfactant hydroxypropyl-β-cyclodextrin (HPCD), strong oxidizer sodium persulfate (Na_2_S_2_O_8_), and Fenton reagent as eluents to repair polluted sediment from the Shenzhen River. These eluents were used individually or in a combined way in order to test the difference of removal efficiency. The results showed that the application of Fenton reagent and sodium persulfate combined eluents (Na_2_S_2_O_8_/Fe^2+^) could achieve effective removal of heavy metals Cu, Cd, Ni, Pb, and Zn in the sediment at the same time, and the efficiency was much higher than a single reagent.

The combination of different eluents in the chemical leaching process can improve the remediation efficiency of heavy metal pollution in river sediments, so that it has good development prospects. However, a large quantity of reagents used in the operation process means high cost. What is worse, the medicament that is not fully utilized will cause secondary pollution to the environment. In addition, the removal efficiency of heavy metals is also different under different conditions such as pH, pollutant concentration, eluent formulation ratio, and processing time (Table 2). Therefore, for this remediation technology, it is key to develop and select high-efficiency heavy metal repair eluents and set the most appropriate eluent conditions. The search for chemical eluents with high remediation efficiency, low price, easy degradation, and no secondary pollution poses an important research direction.

#### 2.1.3. Ultrasonic/Microwave–Chemical Combined Remediation

Ultrasonic/microwave–chemical remediation technology is a pretreatment of the river sediment utilizing ultrasonic/microwave. The leaching of heavy metals in sediment can be maximized by adjusting the duration of ultrasonic/microwave treatment, and then the leached heavy metals will be further treated with chemical regents. He et al. [35] found that, compared with mechanical vibration, ultrasound enhanced the release of Hg to the water phase in sediments. In the presence of algae, the removal rate of Hg^2+^ within 15 min of ultrasound was the same as 60 min of mechanical vibration. This is because the ultrasonic cavitation destroys the floc structure of the sludge and releases intracellular material from the cell wall, which significantly changes the characteristics of both the dissolved state and particulate matter in the liquid. Therefore, the fixed heavy metals attached to the sludge particles and the floc surface are redissolved, increasing the removal rate of heavy metals. Zhu et al. [36] used composite chemical reagents to remove heavy metals Cu, Zn, Mn, Cr, and Ni from sediment, meanwhile carrying out the pre-treatment with ultrasonic and microwave, respectively prior to the experiment. Their results showed that the extraction rate of heavy metals in sediment by combined reagents was significantly improved by the ultrasonic and microwave treatment. Moreover, these two physical treatment methods had different optimal treatment time and power, depending on the power density curve. Wu et al. [37] studied the extraction and separation effect of heavy metal ions in sediment under the assistance of ultrasonic wave. In their study, Cu–Cr and Cr–Fe sediments were respectively removed by a two-stage method with the assistance of ultrasonic waves. Their results showed that, for the Cu–Cr sediment, 96.52% of Cu was leached into the solution while 96.64% of Cr was retained in the leach residue; for the Cr–Fe sediment, 97.05% of Cr was leached into the solution while 98.48% of Fe was retained in the leach residue. Compared with the acid leach method alone, this combined method had obviously higher extraction rates (extraction rates: Cu 97.47% and Fe 1.81% in the Cu–Fe sediment).

The principle of ultrasonic/microwave includes ultrasonic cavitation, mechanical movement, and generation of free radicals. The former two have a strengthening effect on the extraction of heavy metals, while the latter has a significant effect on accelerating the decomposition of refractory organic pollutants. Ultrasound/microwave can also enhance the mass transfer process from solution to adsorbent. Ultrasonic/microwave interacts with the medium during the propagation process, leading to the phase and amplitude change. Therefore, some physical, chemical, and biological properties or states of the medium were changed, resulting in a series of effects including mechanical, thermal, chemical, and biological effects [38]. The main factors influencing the ultrasonic/microwave effect include the ultrasound action time, water content of the sample, and the sound intensity (Table 3). Setting reasonable ultrasonic/microwave processing conditions will significantly improve the repair efficiency.

Ultrasonic/microwave–chemical combined remediation improves the extraction efficiency of heavy metals and reduces the use of chemical agents so as to reduce the risk of secondary pollution to the environment. With advantages of high treatment speed, good remediation effect, mild reaction conditions, and the like, it shows a promising application prospect. However, this technology is currently at an initial stage, and further systematization and industrialization is urgently needed. Accordingly, it is necessary to develop efficient and durable reactors and optimize the use of ultrasonic waves under different conditions.

### 2.2. Biological-Chemical Combined Remediation

#### 2.2.1. Phyto–Stabilizing Agent Combined Remediation

The phytoremediation mainly uses plants to concentrate and remove heavy metals through phytoabsorption, phytoextraction, phytovolatilization, phytostabilization, and rhizofiltration [43,44,45]. There are many heavy metal binding sites in plant root cells, which can absorb and enrich heavy metals, and store them in cell walls and vacuoles. Therefore, the toxicity of heavy metals to themselves is reduced. Super-enriched plants are ideal plants for repairing heavy metal pollution. Heavy metals are removed mainly through the extraction of roots, changes of the root environment, assistance of carriers, efficient transfer of root xylem, chemical sedimentation, physical sedimentation, etc. [46]. As early as 1983, American scientists Chaney et al. [47] proposed the idea of removing heavy metals by using plants that can accumulate them. In addition, British scientists Baker et al. [48] stated the practical possibility of removing and recovering heavy metals by hyperaccumulators. Compared with other methods, phytoremediation has the following irreplaceable advantages: lasting effect, low cost, simple post-treatment, good environmental aesthetics, operable heavy metal recovery, less environment disturbance in the treatment process, and generally no secondary pollution [49]. For remediation of heavy metals with plants, it is a key to find out tolerant hyperaccumulators. However, hyperaccumulators usually grow slowly, have lower biomass relatively, and are selective for heavy metals, making them not suitable for treatment of combined pollution with multiple heavy metals. The above shortcoming limits the application of phytoremediation [50]. To improve the removal efficiency of heavy metals in the process of phytoremediation, researchers used the phytochemical method combining chemical remediation by adding chemical modifiers in the process of plant growth.

The chemical stabilizing agent can change the physical and chemical properties of heavy metals in sediment, then change the existing forms of heavy metals in the sediment through adsorption or coprecipitation. Through this process, the available content of heavy metals is reduced and the mobility of heavy metals in the sediment is inhibited for the purpose of remediating heavy metal pollutions in sediment. Based on the existing research, organic acids and minerals have high stabilizing efficiency for heavy metals in sediment [51,52]. At present, hydroxyapatite (HAP), one of the most commonly used chemical stabilizing agents, can directly inhibit the mobility of heavy metals by virtue of its own absorbability. The cations in its structure can exchange with other ions to reduce the activity and availability of heavy metal ions [53]. In addition, hydroxyl groups in HAP can increase the pH of sediment environment as well as enhance the activity of enzymes. Consequently, the removal of heavy metals by plants was improved [54]. Targeting the severe heavy metal polluted sediments in a river in Zibo, Huang [55] analyzed the removal efficiency of heavy metal polluted sediment using HAP combined with four plant species (i.e., Water mint, Cattails, Maidenhair and Iris). The results showed that the addition of appropriate amounts of HAP could obviously improve the effect of phytoremediation of heavy metal polluted sediments. However, excessive stabilizing agent may reduce the effect of phytoremediation. It is worth noting that this non-permanent measure just changed the existing form of heavy metals in the sediment, heavy metals remain in the sediment, and the stabilized heavy metals may be reactivated with the passage of time, thus having potential risks of secondary pollution.

#### 2.2.2. Phyto–Activator Combined Remediation

Addition of chemical activators can effectively promote the phytoabsorption and phytoextraction of heavy metals by plants. With the reaction of chemical activators, more heavy metals desorb from the surface of sediment particles and transform into more soluble states, increasing the available content of heavy metals in the sediment. As a result, these activators significantly improve the efficiency of phytoremediation of heavy metal pollution. The common types of activators include low-molecular-weight organic acids, chelating agents, and surfactants. The principles of their activation and extraction for heavy metals are basically the same as those of acid leaching, complexing agent leaching, and surfactant leaching in chemical leaching technology. Therefore, there is still a risk of secondary pollution caused by improper control over the addition of chemicals. EDTA is a widely used chelating agent that can chelate with a variety of heavy metals. Due to its poor biodegradability, it is possible to cause environmental risks when applied in the sediment [56]. In addition, previous researches have shown that the capability of the surfactant to activate heavy metals is much higher than that of phytoextraction. Nevertheless, application of surfactants in the process of phytoremediation of heavy metals will inevitably cause secondary pollution while enhancing the phytoremediation efficiency. Compared with the chelating agents and surfactants, low-molecular-weight organic acids are more widely used in practical processes due to their strong activation capability, obvious heavy metal removal effect, and environmental friendliness. Lin et al. [57] used citric acid, glutamic acid N,N-diacetic acid (GLDA), iminodisuccinic acid (IDS), and saponin to study the effect of their addition amounts on Mn extraction from Chinese fir. The results showed that the application of activators enhanced the Mn removal efficiency of Chinese fir while promoting its growth. The Mn removal efficiency was the highest when the above four low-molecular-weight organic acids were combined and applied simultaneously. Ma et al. [58] explored the effect of citric acid, oxalic acid, and acetic acid on the remediation of lead pollution by wild amaranth plants through indoor pot experiments. The results showed that appropriate addition of organic acid significantly changed the content of Pb in wild amaranth plants and enhanced the activity of Pb, thus improving the removal capability of plants. Therefore, the combination of the activation function of low molecular weight organic acids with the extraction characteristics of plants has a promising future for the treatment of heavy metal polluted sediments.

### 2.3. Combined Inter-Organismal Remediation

#### 2.3.1. Phyto–Microorganism Combined Remediation

Microbial remediation refers to the technology of adding microbial or biological growth promoters to polluted sediment for removing toxic metal pollution or recovering economically valuable metals [59]. There are many related researches about the remediation of organic pollutants by microbes in the early stage, but researches and applications focusing on the remediation of heavy metals are relatively less. In recent years, with deepening research on the effect of microbes in the plant rhizosphere, microbial remediation also has attracted more attention. Although microbes cannot directly degrade heavy metals in the sediment, their metabolism production, including organic acids, surfactants, siderophores, chelating agents as well as their redox actions, can activate or fix heavy metals and improve the remediation efficiency of plants against heavy metal pollution. In the process of plant–microbe joint repair, the action mechanism of microorganisms is mainly reflected in two aspects. On the one hand, microorganisms can regulate the absorption and accumulation of heavy metals by plants by changing the form of heavy metals and expanding the extension range of plant roots in order to promote the extraction of heavy metals by plants and enhance the removal of heavy metals in the sediment. On the other hand, the compartmentalization of heavy metals in microbial cells, chelation, and fixation of metabolites can reduce the ability of heavy metals to migrate, thereby enhancing the plant’s ability to retain heavy metals. At the same time, the growth of plant roots secrete protein, sugar, and other organic matter that can be used as nutrient and energy sources of microorganisms, greatly improving the bioavailability and activity of microorganisms. Reciprocity and mutual benefit form a symbiotic body, which strengthens their respective roles in the heavy metal contaminated sediment repair process and improves the removal efficiency of heavy metals in sediment. Li et al. [60] conducted pot experiments by maize–ryegrass intercropping with arbuscular mycorrhizae (AM) fungi inoculated to study the effect of phyto–AM fungi on the remediation of river sediment. The results showed that the absorption rate of Cd by plants significantly increased with the aid of AM fungi, which effectively promoted the activation of the ryegrass for Cd element. Wu et al. [61] used alfalfa and indigenous microorganisms for remediation of the heavy metal polluted river sediment. The results showed that the heavy metals in the sediment were effectively removed; Zn, Ni, Cr, Cu, and Pb were accumulated in the root of alfalfa, and Mn was accumulated in the leaves. During the remediation process, root microorganisms also played a repairing and reinforcing effect role. All of these conditions improved the remediation efficiency of heavy metals in the sediment.

The phyto–microorganism combined remediation method is a comparatively ideal remediation method for having certain ecological and economic benefits including low cost and no secondary pollution. However, microorganisms are easy to mutate under the influence of the environment. At the same time, hyperaccumulators have selective adsorption of heavy metals, which are relatively simple in diversity and limited in cumulative capacity. Therefore, potential research directions should seek to improve the efficiency of microbial remediation, adjust the corresponding environmental factors based on the needs of microorganisms as well as seek for or cultivate hyperaccumulators that absorb a variety of heavy metals.

#### 2.3.2. Phyto–Animal Combined Remediation

Animal remediation mainly employs a series of life activities such as swallowing, peristalsis, and excretion of benthos such as earthworms, frogs, and shellfishes to promote the remediation process, thus realizing the remediation of the heavy metal polluted sediment by animals. When plants and animals are combined for remediation of the heavy metal polluted sediment, animal activities can form a large number of crisscrossed pore canals in the sediment, increase the porosity of the sediment and gravel layer, and improve the transport rate of water and oxygen in the sediment, thus promoting the growth of plants and root microorganisms [62]. On one hand, animals’ secretions can increase the content of available nutrients in sediment, optimizing the living environment of plants so as to promote the growth of plants. On the other hand, a large number of microorganisms in the secretions are also conducive to the remediation process. In addition, the life activities of animals will also affect the pH value of the sediment environment, causing changes in the occurrence forms of heavy metals in the sediment. As a result, the enrichment of heavy metals by plants was promoted with the assistance of animals. Bai [63] proposed the Eisenia foetida–Chlorophytum combined remediation technology for treatment of heavy metals in the excess sludge based on abundant experiments. The experiments showed that earthworms could change the physical and chemical properties of sludge, promote the growth of Chlorophytum, and enhance the capability of Chlorophytum to absorb heavy metals in sludge. To the sludge with different concentrations of Cu, Zn, and Pb, the maximum accumulation amount of these three heavy metals in Chlorophytum were up to 8.03, 3.47, and 4.81 mg/plant.

Phyto–animal combined remediation has outstanding advantages including obvious effect, environmental friendliness, and low price, leading to its wide use in the actual operation process. Unfortunately, few kinds of animals can be used for the remediation of the heavy metal pollution. Furthermore, most of them have poor adaptability and their growth and reproduction are easily affected by externalities such as temperature, humidity, and pH, which limits the application and development of this remediation technology to a certain extent.

## 3. Research Prospects

Treatment of heavy metals in the sediment by combined remediation technology can effectively improve remediation efficiency, shorten the remediation cycle, and improve the remediation effect. In order to better promote the application of combined remediation methods for heavy metal polluted sediments, the research should be further strengthened in the following aspects:

(1) Seek for or make full use of genetic engineering and other new technologies to cultivate hyperaccumulators with large biomass and strong enrichment capacity that can be used for the remediation of various heavy metals; use microbial technology to screen or develop microbial species that are more conducive to the heavy metal removal and explore the environmental applicability of microorganisms for the sake of improving their remediation availability.

(2) Strengthen the research and development of safe and environmentally-friendly chemical reagents, which not only have a high efficiency on heavy metal removal, but also have better biodegradation, and reduce their environmental risks. More attention should be paid to breaking through the existing technical difficulties and realizing the transformation of heavy metals in polluted sediment to stable precipitate or mineralized substances.

(3) Carry out in-depth research on the mechanism of heavy metal removal by different technologies and try more combinations of various remediation technologies. More efforts shall be spared to get a better efficiency of remediation of heavy metal polluted sediment by optimizing the technology system, reducing the cost, shortening remediation cycle, and avoiding secondary pollution arising from the remediation process.

(4) At present, many remediation technologies are still in the laboratory stage and no remediation projects have been carried out on the actual field. In future work, small and pilot tests of various remediation methods should be strengthened in order to speed up the transformation of these technologies from laboratory to actual engineering application.

## 4. Conclusions 

This review specially summarizes the latest research advances in combined remediation technologies of heavy metal polluted sediment. On account of the heterogeneous nature and compositional complexity of sediment, one single physical-, chemical- or bio- method usually cannot achieve the ideal removal affect. Consequently, combined remediation technologies achieve extensive attention for maximizing advantages of the single method and enhancing removal efficiency. Based on the characteristics and application of existing joint repair technologies, more attention should be paid to absorbing the advantages of various repair technologies in the future. Last but not least, the integration and practice of repair technologies are encouraged to make new effective technologies with advantages in repair efficiency, economic cost, environmental friendliness, and scope of application. 

## Figures and Tables

**Table 1:** Combined electrokinetic remediation (EKR) technologies.

Combined Remediation Technology	Principle	References
EKR-acidification	The acidic cleaning solution neutralizes the OH^−^ produced by the cathode in the electrolytic cell, eliminates the sediment alkalinization phenomenon as well as prevents the precipitation of heavy metals, and finally improves the removal efficiency of heavy metals.	[7,9,10]
EKR-flocculant	After the flocculant pretreatment, the sediment undergoes flocculation, resulting in mud–water separation. The porosity of sediment particles becomes larger, which provides channels for ion migration and improves the removal efficiency of heavy metals.	[8,11,12]
EKR-bioleaching	The microbial activity and acid production during the biological leaching process transform the insoluble metal sulfide to soluble metal sulfate. With the assistance of EKR, the ion migration speed is accelerated and the removal efficiency is improved.	[13,14,15]
EKR-permeable reactive barrier (PRB)	This technology combines the advantages of both EKR and the PRB. Under application of an electric field, metal ions react with the filling material in the PRB during the process of migration to the cathode, which stabilizes the heavy metal ions for further centralized processing.	[16,17,18]
EKR-adsorption	Under the action of the electric field force, heavy metal ions migrate to a specific adsorption area, where in-situ removal of heavy metals is achieved by being adsorbed by an adsorbent.	[19,20,21]
EKR-ion exchange membrane	Selective semi-permeable membranes are used to separate the electrodes from the contaminated soil/sediment, preventing the entry of OH^−^ and H^+^, thereby avoiding the alkalization of the cathode and improving the repair efficiency.	[22,23,24]

**Table 2:** Effect of leaching conditions on heavy metal removal.

Heavy Metal	Condition Variable	Main Results	References
Hg, Cd, Cr, Pb, Zn, Ni	Eluent formulation ratio	When 2% hydrogen peroxide and 42% phosphoric acid were used to remove heavy metals from the remaining sludge, the heavy metal removal rates reached more than 90%.	[30]
Cr	Eluent concentration, contact time, liquid/solid ratio, pH	When the treatment time was 1 h, the ethylenediaminetetraacetic acid (EDTA) concentration was 0.1 mol/L, the soil-liquid mass ratio was 1:10, and the pH was 5, the removal effect was the best. The removal rate of Cr reached as high as 22% and Cr^6+^ was 98.5%.	[31]
Cu, Zn, Cr, Pb, Ni, Mn	Eluent concentration, contact time, liquid/solid ratio, pH	Contact time, liquid/solid ratio, and pH of washing agents had notable influence on removal efficiency. The optimal washing conditions were identified as: concentration of 8.0 g/L, contact time of 24 h, liquid/solid ratio of 20/1, and original pH of washing agent.	[32]
Cu, Zn, Pb, Cd	Liquid/solid ratio, pH	When the molar mass ratios of ethylenediamine *N,N*′-disuccinic acid (EDDS) and ethylenebis (oxyethylenenitrilo) tetraacetic acid (EGTA) to heavy metals were 0.81 and 3.92, the pH was 3.95, potential ecological risk index reduction rate can reach a maximum of 86.05%, and the removal rates of Cu, Zn, Pb, and Cd were 72.48%, 62.40%, 59.25%, and 87.45%, respectively.	[33]
Pb, Cd	Eluent concentration, contact time, pH	With the increase of eluent concentration, the leaching efficiency of Pb and Cd generally showed an upward trend; as the pH of the eluent increased, the eluent rate generally decreased; as the eluent time increased, the eluent rate had three trends: (i) overall increase, (ii) first increase, then decrease, and (iii) no significant change.	[34]

**Table 3:** Effects of ultrasound/microwave conditions on heavy metal removal.

Heavy Metal	Influencing Factors	Main Results	References
Hg	Ultrasound action time	With the increase of the action time, desorption of Hg was firstly strengthened, but the rate was reduced when the time further increased. This was because the Al(OH)_3_ precipitating during ultrasonic process absorbed Hg.	[35]
Cu	Sound intensity	When the ultrasonic wave with a frequency of 50 KHz was applied for 3 h, the repair effect was enhanced with the increase of the ultrasonic sound intensity; when the sound intensity reached 150 V, the enrichment ratio of Cu^2+^ increased by 43%.	[39]
Cd, Pb, Cr	Water content of the sample	Under the action of ultrasound, the soil remediation effect was the best when the water content was 14%; when the water content further increased to 16%, the remediation effect was not significantly improved.	[40]
Ge	Ultrasound action time, sound intensity	When the ultrasonic power was 700 W and reacted at 80 °C for 40 min, the leaching effect of Ge was the highest, reaching 92.7%. When the power continuously increased, the effect became worse.	[41]
Cr, V	Ultrasound action time, sound intensity	When ultrasonic leaching was carried out at 60 °C for 60 min, the leaching rate of Cr and V reached the maximum. Ultrasonic assistance significantly reduced the reaction time and leaching temperature.	[42]
